# Glucose Metabolism as a Potential Therapeutic Target in Cytarabine-Resistant Acute Myeloid Leukemia

**DOI:** 10.3390/pharmaceutics16040442

**Published:** 2024-03-22

**Authors:** Joana Pereira-Vieira, Daniela D. Weber, Sâmia Silva, Catarina Barbosa-Matos, Sara Granja, Rui Manuel Reis, Odília Queirós, Young H. Ko, Barbara Kofler, Margarida Casal, Fátima Baltazar

**Affiliations:** 1 Life and Health Sciences Research Institute (ICVS), School of Medicine, University of Minho, Campus of Gualtar, 4710-057 Braga, Portugal; id8183@alunos.uminho.pt (J.P.-V.); id8801@alunos.uminho.pt (C.B.-M.); saragranja@med.uminho.pt (S.G.); rreis@med.uminho.pt (R.M.R.); 2 ICVS/3B’s—PT Government Associate Laboratory, Braga/Guimarães, Portugal; 3 Research Program for Receptor Biochemistry and Tumor Metabolism, Department of Pediatrics, University Hospital of the Paracelsus Medical University, 5020 Salzburg, Austria; d.weber@salk.at (D.D.W.); b.kofler@salk.at (B.K.); 4 Molecular Oncology Research Center, Barretos Cancer Hospital, Barretos 14784-400, SP, Brazil; samiafrahia@hotmail.com; 5 Department of Pathological, Cytological and Thanatological Anatomy, ESS|P.PORTO, 4200-072 Porto, Portugal; 6 REQUIMTE/LAQV, Escola Superior de Saúde, Instituto Politécnico do Porto, Rua Dr. António Bernardino de Almeida, 4200-072 Porto, Portugal; 7 UNIPRO—Oral Pathology and Rehabilitation Research Unit, University Institute of Health Sciences, CESPU, CRL, 4585-116 Gandra, Portugal; odilia.queiros@iucs.cespu.pt; 8 KoDiscovery, LLC, Institute of Marine and Environmental Technology (IMET) Center, 701 East Pratt Street, Baltimore, MD 21202, USA; youngheeko@kodiscovery.org; 9 Center of Molecular and Environmental Biology (CBMA), University of Minho, 4710-057 Braga, Portugal

**Keywords:** chemoresistance, cytarabine, acute myeloid leukemia, metabolic inhibitors, seahorse, glucose metabolism, 3-bromopyruvate, phenformin

## Abstract

Altered glycolytic metabolism has been associated with chemoresistance in acute myeloid leukemia (AML). However, there are still aspects that need clarification, as well as how to explore these metabolic alterations in therapy. In the present study, we aimed to elucidate the role of glucose metabolism in the acquired resistance of AML cells to cytarabine (Ara-C) and to explore it as a therapeutic target. Resistance was induced by stepwise exposure of AML cells to increasing concentrations of Ara-C. Ara-C-resistant cells were characterized for their growth capacity, genetic alterations, metabolic profile, and sensitivity to different metabolic inhibitors. Ara-C-resistant AML cell lines, KG-1 Ara-R, and MOLM13 Ara-R presented different metabolic profiles. KG-1 Ara-R cells exhibited a more pronounced glycolytic phenotype than parental cells, with a weaker acute response to 3-bromopyruvate (3-BP) but higher sensitivity after 48 h. KG-1 Ara-R cells also display increased respiration rates and are more sensitive to phenformin than parental cells. On the other hand, MOLM13 Ara-R cells display a glucose metabolism profile similar to parental cells, as well as sensitivity to glycolytic inhibitors. These results indicate that acquired resistance to Ara-C in AML may involve metabolic adaptations, which can be explored therapeutically in the AML patient setting who developed resistance to therapy.

## 1. Introduction

Acute Myeloid Leukemia (AML) is an aggressive blood cancer that affects the myeloid cell lineage in the bone marrow, characterized by the rapid growth of immature white blood cells, myeloblasts, which compromise the production of normal blood cells [1,2]. The overall survival rates of AML patients are low, especially in patients above 60 years, who are at highest risk [3]. The standard treatment for AML is based on a 7 + 3 chemotherapy regimen, combining anthracyclines and antimetabolite drugs [4,5]. The efficacy of treatment depends on several factors, such as the overall patient health condition, patient age, and the presence of genetic mutations [5]. Targeted therapy has shown promising results in some AML subtypes that present specific gene mutations. AML patients with mutated fms-like tyrosine kinase 3 (*FLT3*) may be considered to receive intensive chemotherapy in combination with the FLT3 inhibitor midostaurin [6]. Patients harboring isocitrate dehydrogenase (*IDH*)*-1* or *IDH-2* mutations may be treated with ivosidenib and enasidenib [7], respectively. More recently, venetoclax, an inhibitor of the antiapoptotic BCL-2 protein, became available for AML patients above 75 years or for patients with other medical conditions that prohibit the use of intensive chemotherapy [8]. Even though targeted therapy is frequently used, conventional chemotherapy is still used in most AML patients.

Despite advances in AML treatment, relapse occurs in about 50% of patients who achieved remission after initial treatment and can occur from a few months to several years after treatment. Also, the survival of AML patients’ post-relapse is dismal [9]. The 5-year overall survival (OS) of relapsed patients is around 10%, and factors such as age, cytogenetics at diagnosis, duration of first complete remission, and undergoing allogeneic transplantation are associated with OS from relapse [3]. Several mechanisms have been described as potential causes of relapse, including the existence of subclones that are present at diagnosis and survive treatment and clonal evolution from leukemic hematopoietic stem cells, as well as the pre-existence or acquisition of genetic mutations that result in drug insensitivity and, consequently, refractory response to treatment [10,11].

Metabolic reprogramming is a recognized hallmark of cancer [12]. Altered metabolism may induce resistance to chemotherapy in cancer cells by increasing energy production and drug efflux, decreasing drug-induced apoptosis, and/or activating proliferative signaling pathways [13]. *IDH-1* and *IDH-2* enzyme mutations lead to overproduction of the oncometabolite 2-hydroxyglutarate (2-HG), which interferes with cell metabolism and epigenetic regulation [14,15]. Additionally, AML cells present a strong dependency on glucose to maintain the increased activity of the pentose phosphate pathway (PPP) and nucleotide biosynthesis [16]. It has also been reported that enhanced glycolysis in AML cells contributes to reduced sensitivity to chemotherapy [17]. Moreover, a prognostic biomarker signature consisting of six glucose metabolism-associated metabolites, namely glycerol-3-phosphate, pyruvate, lactate, 2-oxoglutarate, citrate, and 2-HG, was identified in AML patients [17].

Otto Warburg’s statement that tumor cells preferably use glycolysis even in the presence of oxygen due to mitochondrial dysfunction is outdated [18]. Current evidence suggests that oxidative phosphorylation (OXPHOS) co-exists with elevated glycolysis in cancer cells and is essential for cell bioenergetics, biosynthesis, signaling, and drug resistance [19,20,21,22]. There has been intensive research in the field of cancer metabolism, including AML, but there are still aspects related to metabolic flexibility, mitochondrial metabolism vs. glycolytic metabolism that need further investigation in the context of chemoresistance. Therefore, our aim was to understand the link between glucose metabolism and Ara-C resistance in AML and whether it could be translated into potential therapeutic strategies.

## 2. Materials and Methods

### 2.1. Cell Lines and Culture Conditions

Acute Myeloid Leukemia (AML) cell lines HL-60, NB-4, MOLM13, and KG-1 were acquired from the DSMZ-German Collection of Microorganisms and Cell Cultures GmbH. Cells were maintained and routinely subcultured in culture flasks at a cell density between 1 × 10^5^ and 1 × 10^6^ cells/mL. Cells were grown in a complete medium consisting of RPMI (PanBiotech, Aidenbach, Germany) supplemented with 10% Fetal Bovine Serum (FBS, PanBiotech) and 1% penicillin/streptomycin (PenStrep, PanBiotech). Cell cultures were maintained in a humidified incubator at 37 °C and a 5% CO_2_ atmosphere. To induce resistance, cells were exposed to increasing concentrations of Cytarabine (Ara-C, C3350000, Sigma, Strasbourg, France) and Daunorubicin (DNR, D0125000, Sigma) for 3–6 months. To obtain enough biomass to perform the assays, cells were grown to high densities, but not exceeding 1 × 10^6^ viable cells/mL and with cell viability up to 90%. Viable cell estimation was performed by adding 1:1 cell suspension to 0.2% trypan blue (sc-216028, Santa Cruz Biotechnology, Dallas, TX, USA), and cells were counted in a Neubauer chamber.

### 2.2. Mutational Profile Analysis

DNA was isolated from parental and resistant AML cell lines using the AllPrep^®^DNA/RNA/Protein Mini kit (Qiagen, Hilden, Germany), following the manufacturer’s instructions. Next sequencing generation (NGS)-based mutation analysis was performed using the Archer Variant Plex^®^ Core Myeloid gene panel (SK0121), which contains SNV/Indel, CNV and Internal tandem duplication (ITD) of 37 key genes with the customization of the genes Sterile Alpha Motif Domain Containing 9 (*SAMD9*) and Sterile Alpha Motif Domain Containing 9 Like (*SAMD9L*), at the Molecular Oncology Research Center, Barretos Cancer Hospital, Brazil. Sequencing data produced by this method was converted to de-multiplexed FASTQs and then processed using Archer Analysis (v6.0). For somatic variant interpretation, the AMP/ASCO/CAP consensus guidelines were used, along with other somatic mutations, to correctly classify these variants [23,24].

### 2.3. Cell Growth Rate Estimation

Growth rate (µ) and doubling time (Td) were determined as previously described [25]. AML-resistant and parental cell lines (2.5 × 10^4^ cells/well) were seeded into 24-well plates in complete RPMI medium (11 mM glucose), as well as in RPMI medium without glucose, supplemented with different glucose concentrations (0, 2.5, 5, and 10 mM). Growth curves were generated using manual cell counting in the Neubauer chamber every 24 h for 4 days. The µ and Td were determined according to the growth curve and line equation determination.

### 2.4. Cell Viability Assay

1 × 10^5^ cells/well were seeded in 24-well plates and exposed to a range of increasing Ara-C concentrations (0.05 to 200 µM), 3-Bromopyruvate (3-BP, 1 to 50 µM), 2-Deoxyglucose (2-DG, 1 to 200 mM) and phenformin (0.25 to 5 mM) for 48 h. Drug vehicles, 0.1% DMSO, and PBS were used as controls. Cells were counted using the Trypan blue assay, and cell viability and IC_50_ values for Ara-C, 3-BP, 2-DG, and phenformin were determined and normalized for control. Each treatment was performed in duplicate in at least three independent experiments.

### 2.5. Extracellular Lactate and Glucose Quantification

Cells were plated in 24-well plates at a density of 5 × 10^5^ cells/well and incubated for 24 h. Cell culture supernatants were collected at 0, 4, 6, 12, and 24 h, and extracellular lactate and glucose were quantified as previously described [26], using the Lactate and Glucose-LQ Colorimetric Assays (Spinreact, Girona, Spain), according to the manufacturer’s instructions. The obtained values were normalized for the total number of cells (determined by the Trypan blue assay) in three independent experiments. Glucose consumption was determined by the difference between glucose levels at timepoint 0 and the other timepoints. Data are expressed as mM/10^6^ cells.

### 2.6. Bioenergetic Measurements (Seahorse Assays)

On the day of the assay, KG-1 and MOLM13 parental and Ara-R cells, respectively, were seeded in poly-L-lysine (0.1 mg/mL; Gibco™ A3890401) coated Seahorse XF96 plates at a density of 5 × 10^4^ viable cells per well in 50 μL XF Assay Medium (Seahorse XF RPMI medium supplemented with 10 mM glucose, 1 mM sodium pyruvate and 2 mM L-glutamine, Seahorse Bioscience, Santa Clara, CA, USA). Cells were centrifuged for 1 min at 200× *g* without brake for cell fixation and incubated at 37 °C in a CO_2_-free incubator for 30 min. Once the cells were attached to the plate, an additional 130 μL XF Assay Medium was added, and cells were again incubated for 30 min at 37 °C without CO_2_. To evaluate mitochondrial function by measuring oxygen consumption rate (OCR) in response to mitochondrial stressors and the glycolytic inhibitor 3-BP in parental as well as resistant cells, the Seahorse XF Cell Mito Stress Test was performed. Therefore, port A was loaded with either medium or 33 µM of 3-BP. Port B was loaded with 2.5 μM oligomycin to inhibit ATP-synthase in order to calculate ATP-linked oxygen consumption, port C with 0.5 μM fluoro-carbonyl cyanide phenylhydrazone (FCCP, uncoupler) to measure maximal respiration when cells are stressed, and port D with 0.5 μM rotenone combined with 0.5 μM antimycin A to completely inhibit mitochondrial respiration. To assess the glycolytic capacity of parental and resistant cells, the Glycolytic Rate Assay was performed; since this assay quantifies the glycolytic proton efflux rate (glycoPER), the rate of extracellular acidification due to glycolysis only. Therefore, port A was loaded with either medium or 33 µM of 3-BP, port B with 0.5 μM rotenone combined with 0.5 μM antimycin A to inhibit mitochondrial respiration and, in turn, to assess compensatory maximal glycolysis, followed by the injection of 50 mM 2-deoxy-glucose (2-DG) through port C to inhibit glycolysis. Each measurement was normalized for the number of cells (pmol/min/10^4^ cells).

### 2.7. Western Blotting

Western analysis was performed in protein lysates as previously described [26]. Briefly, protein lysates were prepared from cultured cells using a lysis buffer (1% (*v*/*v*) Triton X-100 and 1% (*v*/*v*) NP-40 (Sigma) in ultrapure water; 1:7 protease inhibitors cocktail (Roche^®^, Basel, Switzerland) and 1:100 phosphatase inhibitor (Sigma). Cells were collected and washed with PBS and were allowed to lyse in 100μL of lysis buffer for 15 min at 4 °C. Lysates were then homogenized (vortex), centrifuged at high speed, and supernatants were collected. Protein concentration was determined using the Bradford assay (B6916, Sigma). Samples were then separated by 10% SDS/PAGE. Proteins were transferred on nitrocellulose membranes (Amersham Biosciences^®^, Amersham, UK) at 100 V for 90 min. For immunostaining, membranes were blocked with 5% (*w*/*v*) BSA in TBS containing 0.1% (*v*/*v*) Tween-20 (TBS-T). Membranes were incubated with GLUT1 (1:500, ab15309), HKII (1:2000, ab104836), MCT1 (1:500, sc-365501); MCT4 (1:500, sc-376465), LDHA (1:2000, sc-137243) and tubulin (1:20,000, ab6046 or 1:1000, sc-5286) primary antibodies, followed by two washing steps (15 min each) in TBS-Tween-20 and incubation with horseradish peroxidase-conjugated secondary antibodies (1:5000, sc-516102 and sc-2357). Proteins were detected using the ECL Chemiluminescence detection kit (ECL, Western Bright TM Sirius, Advansta, San Jose, CA, USA). Each immunoblot was performed at least three times, and the ones selected for figures are from representative experiments (Azure Biosystems, Dublin, CA, USA).

### 2.8. Statistical Analysis

Statistical analysis was performed using the GraphPad Prism 8.4.2 software. Data are presented as the mean ± standard deviation (SD) from 2 or 3 independent experiments. Differences between groups were considered statistically significant at *p* ≤ 0.05, and a trend was considered at 0.05 < *p* ≤ 0.10.

## 3. Results

### 3.1. Development and Characterization of Cytarabine-Resistant KG-1 and MOLM13 Variants

**Figure 1:**
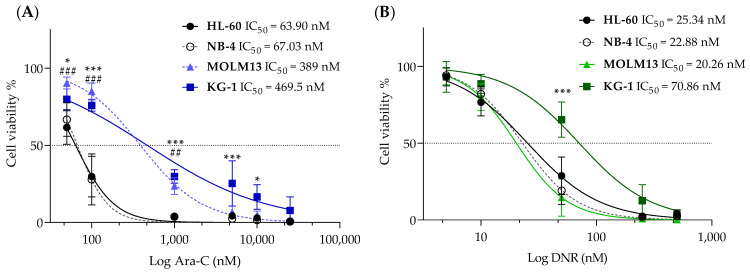
Viability of AML cell lines in response to chemotherapy. Dose-response curves and IC_50_ values for HL-60, NB-4, MOLM13, and KG-1 cell lines treated with (**A**) cytarabine (Ara-C) and (**B**) daunorubicin (DNR). Values are expressed as cell viability relative to vehicle-treated cells normalized to 100%. Values are given as mean ± SD. Two-way ANOVA followed by Sidak’s Multiple Comparison Test: *, *p* ≤ 0.05; ## *p* ≤ 0.01; ***, ### *p* ≤ 0.001. Comparing all cell lines with the KG-1 (*) or the MOLM13 (#) cell line. Results are from at least three independent experiments with two replicates each.

Ara-C and DNR are the standard chemotherapeutic agents given to patients with AML. Thus, we tested the sensitivity of a panel of four AML cell lines (HL-60, NB-4, KG-1, and MOLM13) to Ara-C and DNR (Figure 1). Compared to HL-60 and NB-4, KG-1 and MOLM13 cell lines revealed lower sensitivity to Ara-C, presenting the highest IC_50_ values (>300 nM) (Figure 1A). This difference was not as evident for DNR. HL-60, NB-4, and MOLM13 cells showed IC_50_ values for DNR around 23 nM, while KG-1 displayed a higher IC_50_ value of 71 nM (Figure 1B). Both HL-60 and NB-4 are representative of the acute promyelocytic leukemia subtype, which presents a good prognosis and effective targeted therapy (e.g., all-trans retinoic acid-ATRA). Thus, we selected KG-1 and MOLM13, a myeloid and monocytic cell line, respectively, to induce resistance to Ara-C.

**Figure 2:**
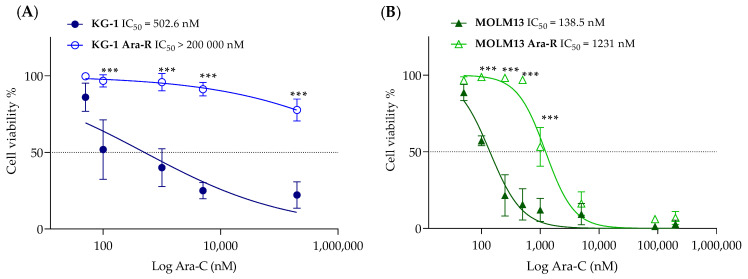
Dose–response curves and determination of the IC_50_ values of cytarabine (Ara-C) in (**A**) KG-1 and (**B**) MOLM13 parental and resistant cell lines. Values are expressed as cell viability relative to vehicle-treated cells normalized to 100%. Values are given as mean ± SD. Two-way ANOVA followed by Sidak’s Multiple Comparison Test: *** *p* ≤ 0.001. Results are from at least three independent experiments with two replicates each.

KG-1 and MOLM13 Ara-C-resistant variants were generated by stepwise exposure to Ara-C for 3–6 months (Figure S1). The two established AML-resistant cells were named KG-1 Ara-R and MOLM13 Ara-R. After the establishment of resistance, IC_50_ values were determined for the parental and resistant cell lines (Figure 2). The range of Ara-C concentrations used for KG-1 cells was not sufficient to determine the IC_50_ value for KG-1 Ara-R cells (for 200 µM Ara-C, about 75% of cells were still viable) (Figure 2A). On the other hand, the IC_50_ value for MOLM13 Ara-R cells was possible to determine, being about 10-fold higher than for MOLM13 parental cells (Figure 2B).

**Figure 3:**
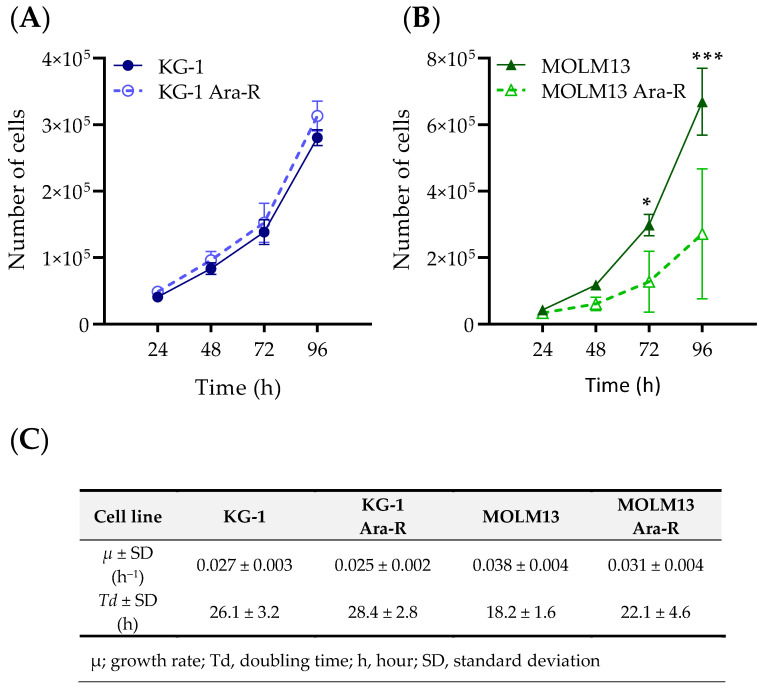
Cell growth rates and doubling times of AML Ara-R resistant and parental cell lines. Cell growth curves of (**A**) KG-1 and KG-1 Ara-R and (**B**) MOLM13 and MOLM13 Ara-R were analyzed over multiple population doublings. 2.5 × 10^4^ cells were plated, and cells were counted every 24 h using Trypan blue dye. (**C**) Values of growth rates (µ) and doubling times (*Td*) were calculated from the respective line equations (Figure S2). Statistical significance was determined by two-way ANOVA followed by Sidak’s Multiple Comparison Test. * *p* ≤ 0.05; *** *p* ≤ 0.001. At least three independent experiments with three replicates were performed.

In addition to cell viability, growth rates and doubling times were determined for parental and Ara-C-resistant cells (Figure 3A–C and Figure S2). Comparing parental and Ara-C-resistant cells, no differences in growth rates were observed between KG-1 and KG-1 Ara-R cells (Figure 3A,C), while MOLM13 Ara-R cells grew at a slower rate than MOLM13 parental cells, with doubling times of about 24 h and 18 h, respectively (Figure 3B,C). We also evaluated the effect of the absence and the presence of different glucose concentrations (0, 2.5, 5, and 10 mM) on growth rate and doubling time. In general, the growth rates of both cell line pairs increased as glucose concentrations rose. No significant differences were observed in growth rates between parental and KG-1 Ara-R cells, but MOLM13 Ara-R grew faster than parental cells in the absence of glucose (Figure S3). To assess the capacity to maintain the resistance phenotype, we cultured Ara-C-resistant cells without Ara-C for 3 weeks, which did not result in the loss of Ara-C resistance (Figure S4). This indicates that Ara-C resistance was not reversible over time in these cell lines. Additionally, we assessed if Ara-C-resistant cells were also resistant to the DNR (Figure S5). KG-1 Ara-R cells were significantly less sensitive to DNR (higher IC_50_ values) when compared to parental cells. However, no differences were observed for MOLM13 cells, which suggests different mechanisms of resistance in the two cell lines.

NGS-based mutation analysis of Ara-C resistant and parental cells identified a total of fourteen variants in ten genes frequently mutated in AML patients (Table 1) [27]. Five of the fourteen mutations found are described in the catalog of somatic mutations in cancer (COSMICs). According to the AMP/ASCO/CAP consensus guidelines [23,24], it was possible to classify one as Variants of Strong Clinical Significance (Tier I), one as Variants of Potential Clinical Significance (Tier II), four as Variants of Unknown Clinical Significance (Tier III) and eight as Benign or Likely Benign Variants (Tier IV). Comparing parental and Ara-C resistant cells, the differences found were loss of the *NRAS* mutation in KG-1 Ara-R cells, whereas MOLM13 Ara-R acquired an additional *CEBPA* variant mutation.

### 3.2. Characterization of the Glycolytic and Respiratory Profile of Parental and Ara-R AML Cell Lines

**Figure 4:**
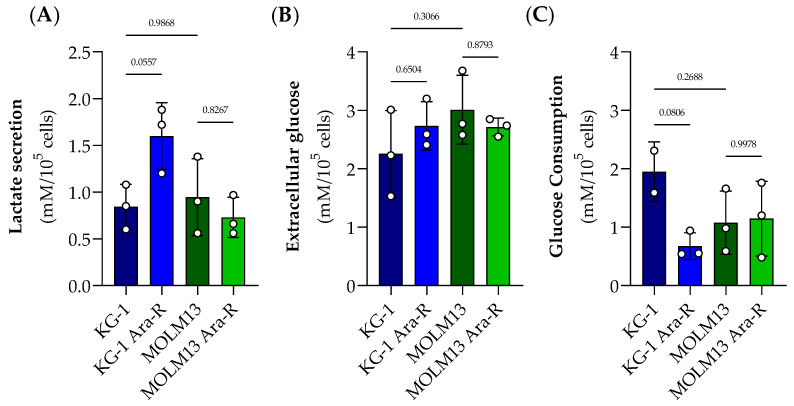
Lactate secretion and glucose consumption in parental and Ara-R cell lines. (**A**) Levels of lactate secretion and (**B**) extracellular glucose were evaluated at 4 h for KG-1, KG-1 Ara-R, MOLM13, and MOLM13 Ara-R cells. (**C**) Glucose consumption corresponds to the difference in glucose concentration between 0 h and 4 h of incubation (Figure S6). Results are presented as mean ± SD of at least three independent experiments. Statistical significance estimated by two-way ANOVA followed by Sidak’s Multiple Comparison Test.

The *FLT3*, *RAS*, and *TP53* genes are involved in cell metabolic regulation of glycolysis. As a first approach to identify metabolic alterations in glucose metabolism associated with Ara-C resistance, we assessed glucose consumption and lactate secretion to the medium of parental and resistant cells at different time points (Figure S6). However, no significant differences were observed between Ara-C resistant and parental cells for both cell lines. Nevertheless, at 4 h, KG-1 Ara-R cells showed a trend to increase in lactate secretion compared to KG-1 parental cells (Figure 4A), but no difference was observed in extracellular glucose levels (Figure 4B). When calculating glucose consumption, KG-1 Ara-R cells tended to consume glucose slower than KG-1 parental cells (Figure 4C). For MOLM13 cells, no significant differences were observed between parental and resistant cells either for lactate secretion (Figure 4A) or glucose consumption (Figure 4C).

**Figure 5:**
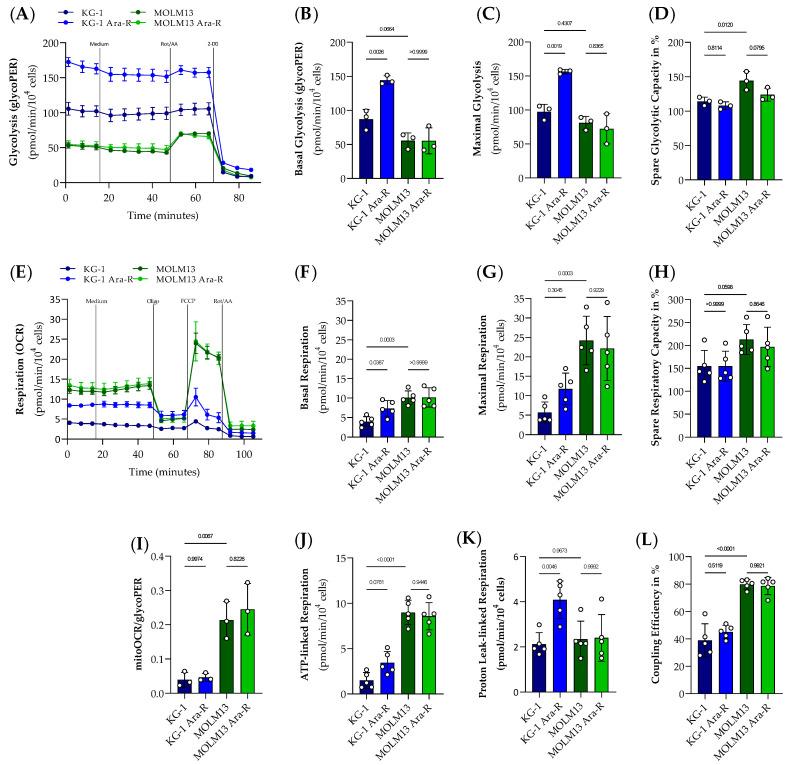
Characterization of the glycolytic and respiratory profile in parental and Ara-R cell lines. Results of the Glycolytic rate (**A**) and Mito Stress (**E**) test in KG-1, KG-1 Ara-R, MOLM13, and MOLM13 Ara-R cells are presented as real-time measurements of glycolytic proton efflux rate (glycoPER) and oxygen consumption rate (OCR) normalized to cell number, respectively. (**B**) Basal glycolysis, (**C**) maximal glycolysis, (**D**) glycolytic reverse in %, (**F**) basal respiration, (**G**) maximal respiration, (**H**) spare respiratory capacity in %, (**I**) ratio of respiration to glycolysis, (**J**) ATP-linked respiration, (**K**) proton leak-linked respiration, (**L**) coupling efficiency in %. Values are given as mean ± SD. One-way ANOVA followed by Sidak’s Multiple Comparison Test. At least three independent measurements with 2 to 8 replicates were performed for each cell line. Treatments: 2.5 µM Oligomycin (Oligo.); 0.5 µM fluoro-carbonyl cyanide phenylhydrazone (FCCP); 0.5 µM Rotenone and Antimycin A (Rot/AA); 50 mM 2-Deoxy-d-glucose (2DG).

Next, we measured the glycolytic proton efflux rate (glycoPER) and oxygen consumption rate (OCR), indicators of glycolytic activity and mitochondrial function, respectively, to compare the glycolytic and respiratory profile between KG-1 and MOLM13 cells as well as between parental and Ara-C resistant cell lines (Figure 5).

Resistance to Ara-C increased basal glycolysis in KG-1 but not in MOLM13 cells. This result is in accordance with the observed increase in lactate secretion at 4 h in KG-1 Ara-R but not MOLM13 Ara-R cells compared to the respective parental cells. The comparison of both parental AML cell lines showed a trend to lower basal glycolysis in MOLM13 compared to KG-1 cells (Figure 5A,B). The ability of cells to compensate for energy production through glycolysis after inhibiting mitochondrial respiration (maximal glycolysis) was also increased by Ara-C resistance in the KG-1 but not in the MOLM13 cell line (Figure 5C). The glycolytic reserve (i.e., maximal/basal glycolysis), which indicates how close the glycolytic function is to the cell’s theoretical maximum, did not differ between KG-1 parental and resistant cells but tended to be lower in MOLM13 Ara-R compared to MOLM13 cells. Moreover, MOLM13 cells showed a higher glycolytic reserve compared to KG-1 cells (Figure 5D).

In terms of respiration, Ara-C resistance increased basal as well as proton-leak-linked respiration and tended to increase ATP-linked respiration in KG-1 cells but not in MOLM13 cells (Figure 5E,F,J,K). However, resistance to Ara-C did not affect maximal respiration, spare respiratory capacity (i.e., maximal/basal respiration), and coupling efficiency (i.e., ATP-linked/basal respiration) (Figure 5G,H,L). Basal and maximal respiration, as well as ATP-linked respiration and coupling efficiency, were higher, whereas spare respiratory capacity tended to be higher in MOLM13 compared to KG-1 cells (Figure 5E–H,J,L).

Since MOLM13 cells presented lower glycolysis and higher respiration rates compared to KG-1 cells, the ratio of respiration to glycolysis (mitoOCR/glycoPER) was also higher in MOLM13 compared to KG-1 cells (Figure 5I). However, the ratio of respiration to glycolysis remained unaffected by Ara-C resistance (Figure 5I) because glycolysis and respiration were both (a) increased by Ara-C resistance in KG-1 cells and (b) not affected by Ara-C resistance in MOLM13 cells.

To check if these metabolic alterations were translated into changes in protein expression, we evaluated the expression of metabolism-related key proteins by Western blot, namely glucose transporter 1 (GLUT1), monocarboxylate transporters 1 and 4 (MCT1/MCT4), as well as the hexokinase II (HKII) and lactate dehydrogenase A (LDHA) enzymes at different timepoints (6 and 24 h). The only difference observed was a significant decrease in LDHA at 24 h in KG-1 Ara-R compared to KG-1 parental cells (Figure S7). This could be explained by the fact that the levels of extracellular glucose were very low at 24 h (Figure S6), and the levels of secreted lactate did not increase after 12 h. Thus, the need for converting pyruvate into lactate might be lower.

### 3.3. Effect of Metabolic Inhibitors on Cytarabine-Resistant Cells

**Figure 6:**
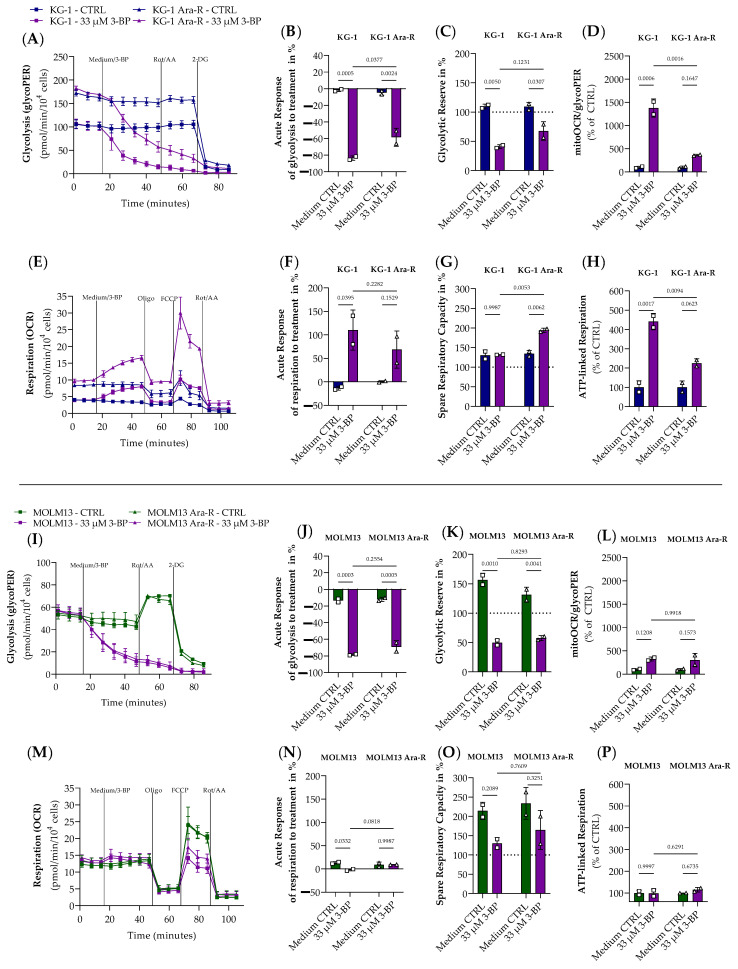
Effect of 3-bromopyruvate (3-BP) on glycolysis and respiration in parental and Ara-R cell lines. Results of the Glycolytic rate (**A**,**I**) and Mito Stress (**E**,**M**) test in (**A**–**H**)) KG-1, KG-1 Ara-R, (**I**–**P**) MOLM13, and MOLM13 Ara-R cells are presented as real-time measurements of glycolytic proton efflux rate (glycoPER) and oxygen consumption rate (OCR) normalized to cell number. (**B**,**J**) acute response of glycolysis to 3-BP, (**C**,**K**) glycolytic reserve in %, (**D**,**L**) ratio of respiration to glycolysis. (**F**,**N**) acute response of respiration to 3-BP, (**G**,**O**) spare respiratory capacity in %, (**H**,**P**) ATP-linked respiration. Values were given as mean ± SD. One-way ANOVA followed by Sidak’s Multiple Comparison Test. Two independent measurements with 2 to 6 replicates were performed for each cell line. Treatments: 2.5 µM Oligomycin (Oligo.); 0.5 µM fluorocarbonyl cyanide phenylhydrazone (FCCP); 0.5 µM Rotenone and Antimycin A (Rot/AA); 50 mM 2-Deoxy-d-glucose (2DG).

Aiming to target the increased glycolytic rate induced by Ara-C resistance in KG-1 cells, the effect of 3-BP, a glycolytic inhibitor, was evaluated in Ara-C-resistant and parental cells using the Seahorse XF Cell Mito Stress and Glycolytic Rate tests (Figure 6).

The acute treatment with 33 μM of 3-BP strongly reduced glycolysis in both KG-1 and KG-1 Ara-R cell lines. Although KG-1 Ara-R cells showed increased glycolysis compared to KG-1 cells, the acute response to 33 µM of 3-BP was relatively stronger in KG-1 compared to KG-1 Ara-R cells (Figure 6A,B). Moreover, 3-BP-treated cells were not able to increase glycolysis after Rot/AA treatment (Figure 6C), representing that 3-BP diminished the glycolytic reserve. As a response to reduced glycolysis induced by 33 µM 3-BP, both KG-1 and KG-1 Ara cells increased respiration (Figure 6E,F). Spare respiratory capacity was increased by 3-BP in KG-1 Ara-R cells compared to medium control treatment as well as between KG-1 Ara-R compared to KG-1 cells both treated with 3-BP (Figure 6G). Relative to the medium control, 3-BP induced a stronger increase in ATP-linked respiration in KG-1 compared to KG-1 Ara cells (Figure 6H). These results are also reflected in the stronger increase in the ratio of respiration to glycolysis in KG-1 cells compared to KG-1 Ara-R cells relative to the respective untreated control (Figure 6D).

3-BP also acutely inhibited glycolysis as well as the glycolytic reserve in MOLM13 and MOLM13 Ara-R cells (Figure 6I–K). However, MOLM13 parental as well as resistant cells were not able to compensate for the reduction in glycolysis by increased respiration, spare respiratory capacity, or ATP-linked respiration (Figure 6M–P). Also, the ratio of respiration to glycolysis remained unaffected by 3-BP treatment in MOLM13 parental and resistant cells (Figure 6L).

**Figure 7:**
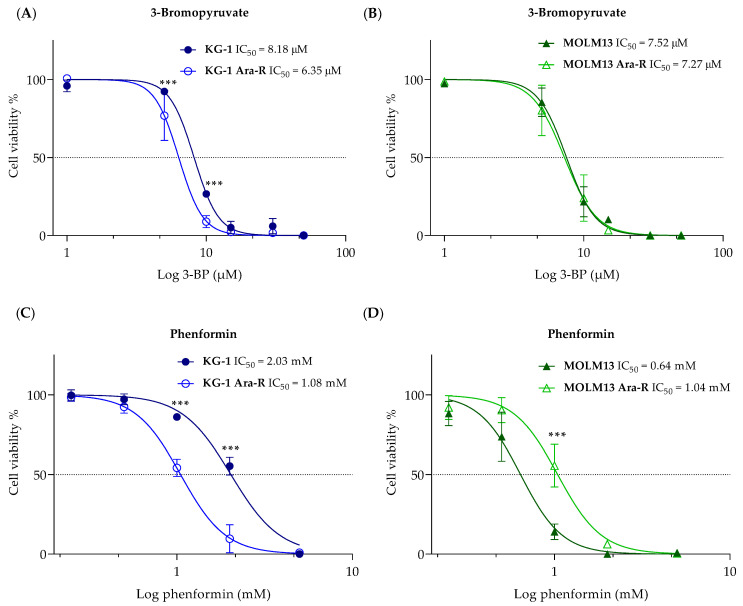
Effect of 3-bromopyruvate (3-BP) and phenformin on Ara-C-resistant and parental cell viability. Dose-response curve to generate IC_50_ values of (**A**,**C**) KG-1 and KG-1 Ara-R cell lines and (**B**,**D**) MOLM13 and MOLM13 Ara-R cell lines in response to (**A**,**B**) 3-BP, and (**C**,**D**) phenformin. Values are expressed as cell viability relative to vehicle-treated cells normalized to 100%. Values are given as mean ± SD. Two-way ANOVA followed by Sidak’s Multiple Comparison Test. *** *p* < 0.001. At least three independent experiments with two replicates were performed.

Next, we evaluated the effect of 3-BP on cell viability of both KG-1 and MOLM13 parental and Ara-R cells after 48 h, respectively (Figure 7). KG-1 Ara-R cells were more sensitive to 3-BP, with a significant decrease in cell viability compared to the KG-1 cells (Figure 7A), which is in line with the observed increased glycolytic activity induced by Ara-C resistance in KG-1 cells. For MOLM13 Ara-R and parental MOLM13 cells, no difference was observed in cell viability after 3-BP exposure (Figure 7B). Additionally, we tested another glycolytic inhibitor, 2-DG, and the respiration inhibitor, phenformin. 2-DG induced a similar effect to 3-BP in both KG-1 cell lines (higher sensitivity for Ara-C-resistant cells) and MOLM13 cell lines (no difference) (Figure S8). For phenformin treatment, KG-1 Ara-R cells exhibited higher sensitivity than parental KG-1 cells, while MOLM13 Ara-R were less sensitive to phenformin than MOLM13 cells (Figure 7C,D). Overall, KG-1 Ara-R cells displayed increased respiration levels (Figure 5), which might explain the increased sensitivity to phenformin in this cell line.

## 4. Discussion

Different recurrent gene mutations have been identified in AML [28]. The established Ara-C-resistant cell lines showed similar mutation profiles as the parental cell lines, including mutations in *DDX41*, *CEBPA*, *ASXL1*, *SAMD9*, *SAMD9L*, *FLT3*, *NRAS*, and *TP53*. The diagnostic and prognostic value of those mutations was already reported in AML patients, as well as their involvement in cellular processes such as differentiation, proliferation, and cell death in AML [2,28,29]. CEBPA mutations are one the most frequent genetic alterations in AML patients. CEBPA is a transcription factor, controlling gene expressions responsible for cell proliferation and differentiation. Double-mutated CEBPA is associated with a favorable prognosis, while single-mutated CEBPA does not seem to improve prognosis [30]. Recent studies have shown that the biallelic CEBPA mutations in AML do not appear to modify prognostic, but the coexistence with other chromosomal abnormalities and gene mutations may influence prognostic [31]. Although the number of somatic mutations present at diagnosis appears to be present at relapse [32,33], our data show that KG-1 Ara-R cells lost *NRAS* mutation. According to the study of Farra et al. [34], most pediatric AML patients with mutated *NRAS* at first diagnosis lose this mutation at relapse [34]. Maybe this loss of *NRAS* mutation is a drug resistance-related alteration, which allows AML cells to survive after induction chemotherapy and make it a dominant clone and cause recurrence. Moreover, *FLT3*, *TP53*, and *NRAS* mutations are classified as driver oncogenes in AML, and co-occurring mutations in those genes have been associated not only with resistance but also with metabolic adaptations [28,35,36,37]. Here, we observed that KG-1 Ara-R cells lost the *NRAS* mutation despite presenting higher levels of glycolysis and respiration compared to KG-1 cells. This issue deserves further investigation.

Our aim was to characterize glucose metabolism in Ara-C-resistant AML cell lines as a means of identifying potential metabolic targets for therapy. Otto Warburg described that even in the presence of oxygen, cancer cells prefer to ferment glucose to lactate rather than to oxidize glucose in the TCA cycle [18]. In AML, beyond the glycolytic phenotype, glucose metabolism has also been linked to pathways, including the pentose-phosphate, amino acid, glutamine, and fatty acid pathways [14,35,38]. Hence, glycolysis does not only generate energy but also serves the purpose of generating molecular building blocks to sustain cancer survival and proliferation [38].

The results of our established AML cells resistant to Ara-C suggest that different glycolytic and respiration profiles could influence the response to different stresses. MOLM13 cells are more dependent on respiration, whereas the corresponding resistant ones have less capacity to resort to glycolysis when exposed to respiratory inhibition (glycolytic reserve). On the other hand, KG-1 Ara-R cells are more glycolytic but also present higher levels of basal and ATP-linked respiration compared to KG-1 parental cells, indicating a higher ATP demand due to Ara-C resistance. The plasticity of cancer cells is frequently observed in terms of metabolic adaptations. For example, cancer cells are able to switch between glycolysis and respiration as well as to fuel these metabolic pathways with other substrates beyond glucose [39]. In AML, a highly diverse and flexible metabolism contributes to the aggressiveness of the disease, as well as drug resistance [40,41,42]. By using different sources of nutrients for energy and biomass supply, AML cells gain metabolic plasticity and outcompete normal hematopoietic cells [39].

Lastly, we explored glucose metabolism as a potential target in AML Ara-R cells. We tested a 3-BP, a derivative of pyruvate, which is an alkylating agent with anti-cancer effects in different in vitro and in vivo cancer models [42]. 3-BP is a substrate of the monocarboxylate transporters (MCTs), and once inside the cell, it blocks glycolysis by targeting HKII and thereby depleting cell energy. HKII inhibition leads to its dissociation from mitochondria and consequently promotes the release of the apoptosis-inducing factor (AIF) and cytochrome c, thus triggering apoptosis [43]. In this study, the acute treatment of cells with 3-BP inhibited glycolysis in KG-1 and MOLM13 parental and Ara-R cells. However, only KG-1 parental and Ara-C-resistant cells were able to compensate for this effect by switching from glycolysis to respiration. Treatment of cells with 3-BP for 48 h, however, reduced cell viability by 100%. The acute treatment with 3-BP may demonstrate that the first target is glycolysis, but given the alkylating nature of 3-BP, other intermediates involved in glucose metabolism may be targeted by 3-BP [42,43,44,45]. Additionally, we tested the effect of the mitochondrial inhibitor phenformin, which inhibits the complex I of the mitochondrial respiratory chain [46,47], and Ara-C resistance induced an increase in respiration in KG-1 Ara-R cells. On the other hand, KG-1 Ara-R cells also presented an increase in proton leakage that could indicate increased damage of mitochondrial membranes or complexes of the electron transport chain, supporting the observed higher sensitivity of KG-1 Ara-R cells to phenformin compared to KG-1 parental cells. In contrast, MOLM13 Ara-R cells were less sensitive to phenformin compared to MOLM13 parental cells. Still, the IC_50_ value for phenformin was about three times lower in MOLM13 compared to KG-1 cells, which corresponds to higher respiration levels observed in MOLM13 cells. In general, AML cells were more sensitive to treatment with 3-BP than phenformin (higher IC_50_ values), which could be explained by the fact that 3-BP may be acting by two mechanisms of action, being more potent than phenformin.

Hence, it is important to consider the remarkable metabolic adaptability of AML cells, which allows them to survive and thrive in toxic environments, such as when exposed to chemotherapy. This highlights the significance of incorporating a metabolic characterization of the malignant cells to guide the selection of therapeutic strategies. In our two Ara-C-resistant cell models, it became evident that the model that presented an enhanced glucose metabolism with a higher glycolytic profile (KG-1 Ara-R cells) was the one that responded better to the metabolic inhibitors. Thus, glycolytic inhibitors should be explored as a strategy to treat Ara-C-resistant AML with enhanced glucose metabolism.

## 5. Conclusions

In the present study, we report, by different methodologies, that MOLM13 and KG-1 parental cell lines have distinct metabolic profiles: MOLM13 cells are more oxidative, and KG-1 cells are more glycolytic. When inducing resistance to Ara-C, MOLM13 Ara-R cells did not change their metabolic profile, and their growth rate was lower than parental cells. This result suggests an arrest in the cell cycle that may be involved in the mechanism of resistance in MOLM13 Ara-R cells. On the other hand, Ara-R resistance in KG-1 cells induced a more pronounced glycolytic profile. KG-1 Ara-R cells display higher extracellular levels of lactate, glycoPER, and OCR. The response of KG-1 Ara-R cells to acute inhibition of glycolysis led to a shift towards respiration, but the treatment with 3-BP for 48 h significantly decreased cell viability, suggesting that the cells could not handle the induced stress at this time point. In summary, we established two AML cell line models of resistance to Ara-C, which involve metabolic adaptations and have different sensitivities to metabolic inhibitors. Thus, modulation of glucose metabolism has the potential to be explored in AML resistance.

## Figures and Tables

**Table 1:** Myeloid gene mutation panel in parental and Ara-R AML cell lines revealed by NGS analysis.

	KG-1	KG-1 Ara-R	COSMIC ID	Clinical Significance ^@^	Consequence
Mutated genes	*BCOR* (c.4886G>A; p.(Trp1663*))	*BCOR* (c.4886G>A; p.(Trp1663*))		Tier III	Nonsense
	*DDX41* (c.27+2_27+5dup)	*DDX41* (c.27+2_27+5dup)		Tier IV	Frameshift
	*FLT3* (c.1669G>A; p.(Val557Ile))	*FLT3* (c.1669G>A; p.(Val557Ile))	COSM28043	Tier IV	Missense
	*NRAS* (c.360G>T; p.(Leu120Phe))	*-*		Tier III	Missense
	*SAMD9* (c.223C>T; p.(Arg75Trp))	*SAMD9* (c.223C>T; p.(Arg75Trp))		Tier IV	Missense
	*SAMD9L* (c.1217G>A; p.(Arg406Gln))	*SAMD9L* (c.1217G>A; p.(Arg406Gln))		Tier IV	Missense
	*TP53* (c.672+1G>A)	*TP53* (c.672+1G>A)	COSM2744696	Tier II	Splice donor
	**MOLM13**	**MOLM13 Ara-R**	**COSMIC ID**	**Clinical Significance ^@^**	**Consequence**
Mutated genes	*ASXL1* (c.1954G>A; p.(Gly652Ser))	*ASXL1* (c.1954G>A; p.(Gly652Ser))	COSM1716555	Tier IV	Missense
	*CBL* (c.1227_1227+13del)	*CBL* (c.1227_1227+13del)		Tier IV	Frameshift
	*CEBPA* (c.584_589dup; p.(His195_Pro196dup))	*CEBPA* (c.584_589dup; p.(His195_Pro196dup))		Tier IV	Frameshift
	*CEBPA* (c.568T>C; p.(Ser190Pro))	*CEBPA* (c.568T>C; p.(Ser190Pro))		Tier III	Missense
	*-*	*CEBPA* (c.566C>A; p.(Pro189His))		Tier III	Missense
	*FLT3* (c.1775_1795dup; p.(Glu598_Tyr599insPheAspPheArgGluTyrGlu) (FLT3-ITD, 21bp)	*FLT3* (c.1775_1795dup; p.(Glu598_Tyr599insPheAspPheArgGluTyrGlu) (FLT3-ITD, 21bp)	COSM849	Tier I	Inframe insertion
	*SAMD9L* (c.866T>C; p.(Phe289Ser))	*SAMD9L* (c.866T>C; p.(Phe289Ser)))	COSM3982291	Tier IV	Missense

^@^ AMP/ASCO/CAP consensos [23,24].

## Data Availability

Data is available on request due to restrictions.

## Supplementary Materials

The following supporting information can be downloaded at https://www.mdpi.com/article/10.3390/pharmaceutics16040442/s1, Figure S1: Calculation of doubling time (Td) and growth rates (µ) in cytarabine (Ara-C)-resistant cell lines; Figure S2: Induction of Ara-C resistance in AML cell lines; Figure S3: Sensitivity of cytarabine (Ara-C)-resistant cells in the absence of Ara-C; Figure S4: Extracellular lactate and glucose, glucose consumption in parental and Ara-C resistant cell lines; Figure S5: Effect of 2-deoxy-glucose (2-DG) on Ara-C-resistant and parental AML cell viability; Figure S6: Extracellular lactate and glucose, glucose consumption in parental and Ara-C resistant cell lines; Figure S7: Characterization of glycolytic phenotype-associated protein expression in cytarabine (Ara-C)-resistant cell lines; Figure S8: Effect of 2-deoxy-glucose (2-DG) on Ara-C-resistant and parental AML cell viability
