# Supplementary material for: Glucose Metabolism as a Potential Therapeutic Target in Cytarabine-Resistant Acute Myeloid Leukemia
Source: Pharmaceutics. 2024 Mar 22;16(4):442. doi: 10.3390/pharmaceutics16040442 (PMC11055074; doi:10.3390/pharmaceutics16040442)
Supplement: Supplementary file 1 [file pharmaceutics-16-00442-s001.zip › pharmaceutics-2926530-supplementary.pdf]

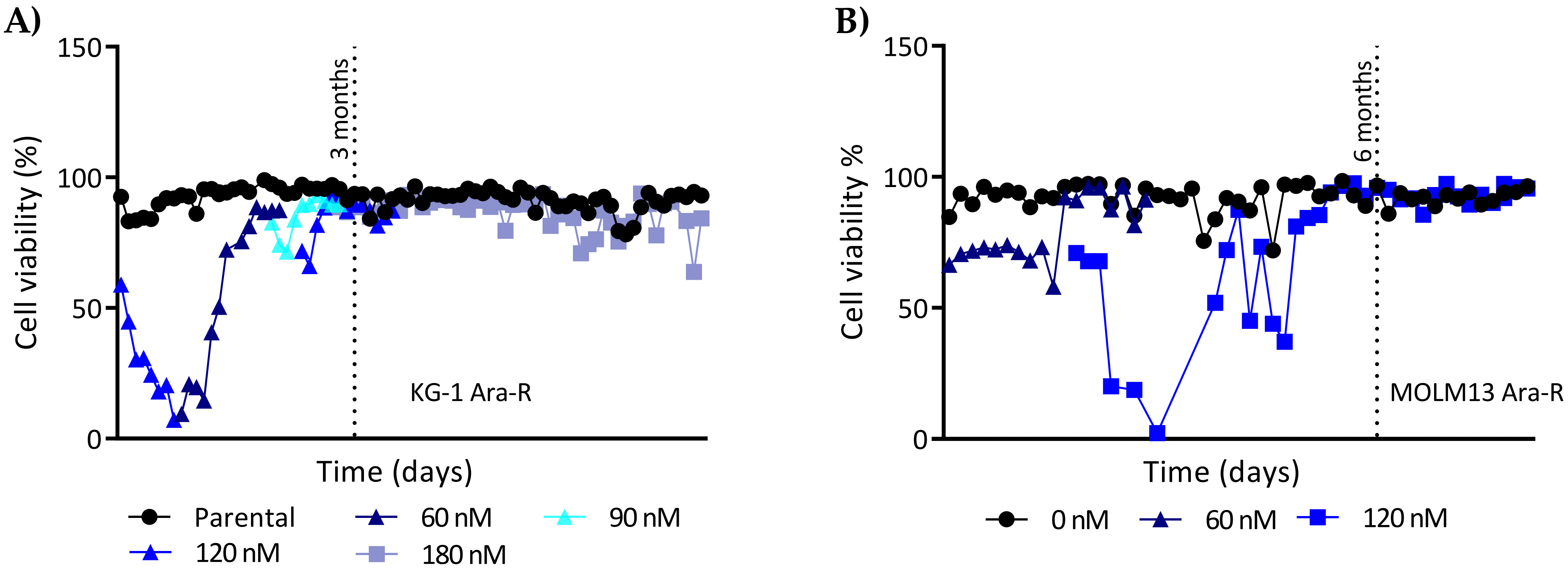

Figure S1. Induction of Ara-C resistance in AML cell lines. (A) KG-1 and (B) MOLM13 cells were exposed to increasing concentrations of cytarabine (Ara-C). Resistance to Ara-C was induced in KG-1 and MOLM13 cells for 3 and 6 months, respectively.

A)

Number of cells (mean of four independent experiments)				
**Cell line**	**KG-1**		**KG-1 Ara-R**	
**Time** (h)	MEAN	SD ±	MEAN	SD ±
**24**	36979	10225	42813	19153
**48**	74583	18887	78958	35929
**72**	133125	18576	126979	56648
**96**	256979	47888	258021	111830
**Td** = ln(2)/**B** (h)	26.058		28.408	
**μ** = [ln(N(96)/N(24))]/t(72) (h^{-1})	0.027		0.025	

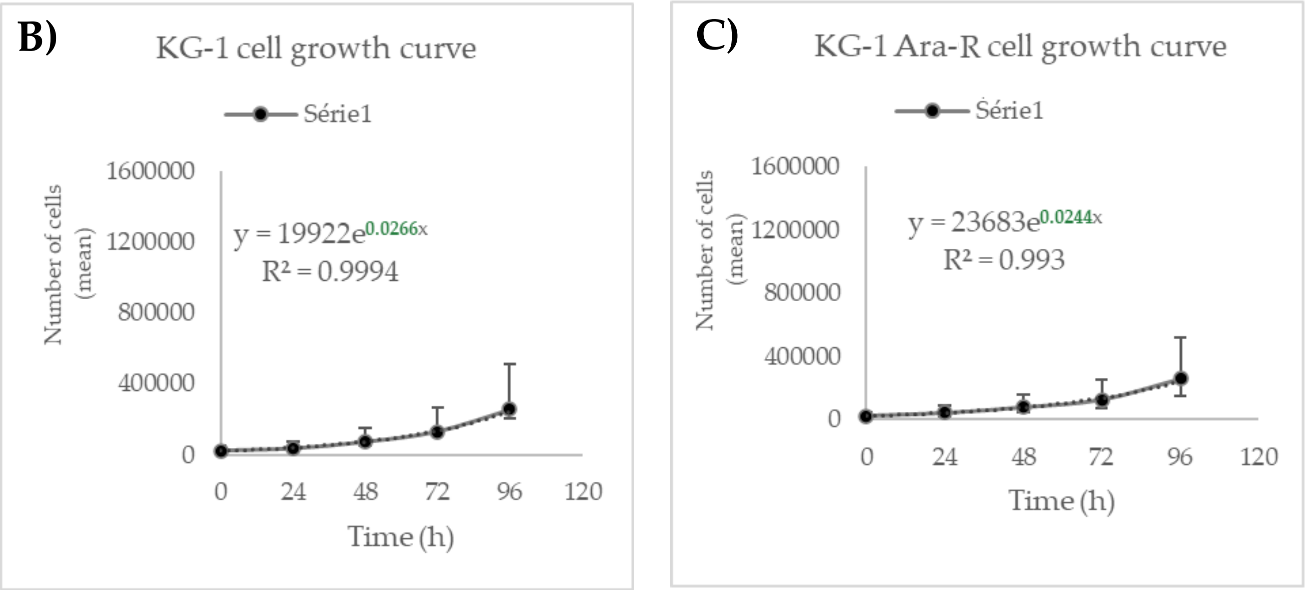

D)

Number of cells (mean of four independent experiments)				
**Cell line**	**MOLM13**		**MOLM13 Ara-R**	
**Time** (h)	MEAN	SD ±	MEAN	SD ±
**24**	43125	8344	34444	14181
**48**	117813	12348	74861	35607
**72**	298229	32103	159167	123627
**96**	668750	100606	326389	248965
**Td** = ln(2)/**B** (h)	18.193		22.145	
**μ** = [ln(N(96)/N(24))]/t(72) (h^{-1})	0.038		0.031	

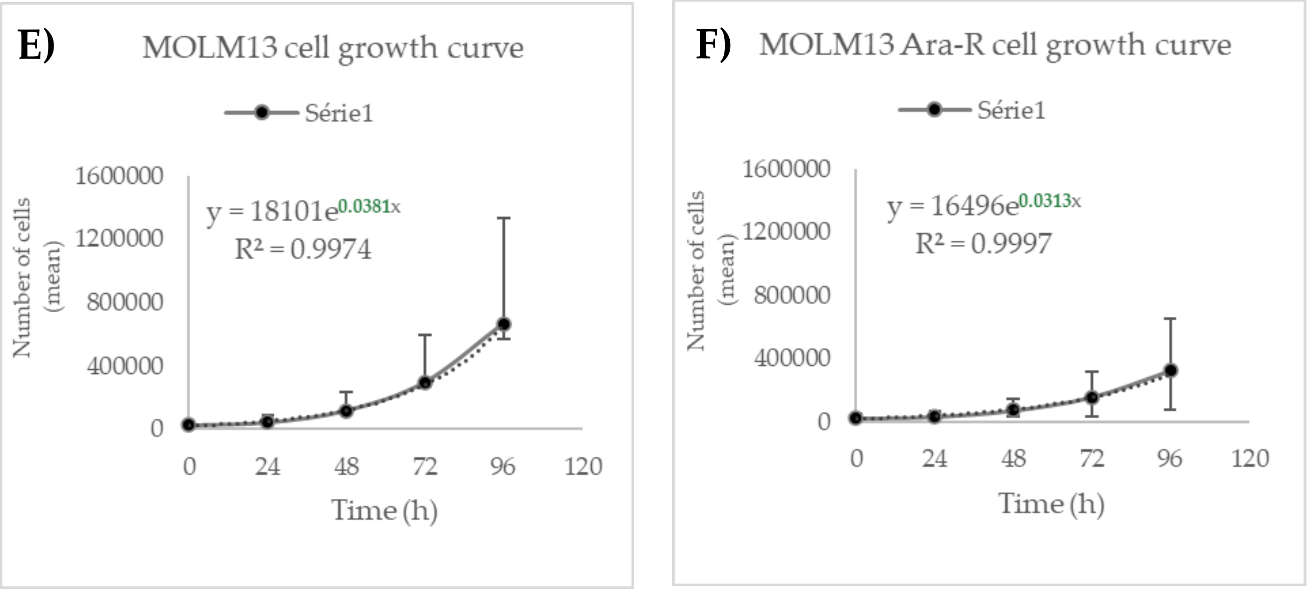

Figure S2. Calculation of doubling time (Td) and growth rates (μ) in cytarabine (Ara-C)-resistant cell lines. A-C) KG-1, KG-1 Ara-R and D-F) MOLM13, MOLM13 Ara-R cell lines were seeded into 24-well plates in complete RPMI medium. B-C, E-F) Growth curves were generated from mean of cell numbers of four independent experiments shown in A-C) for KG-1 and KG-1 Ara-R cells, and in D-E) for MOLM13 and MOLM13 Ara-R cells, using manual cell counting in the Neubauer chamber every 24h for 4 days. Td was calculated from line equation were the green values corresponded to the B value.

A)

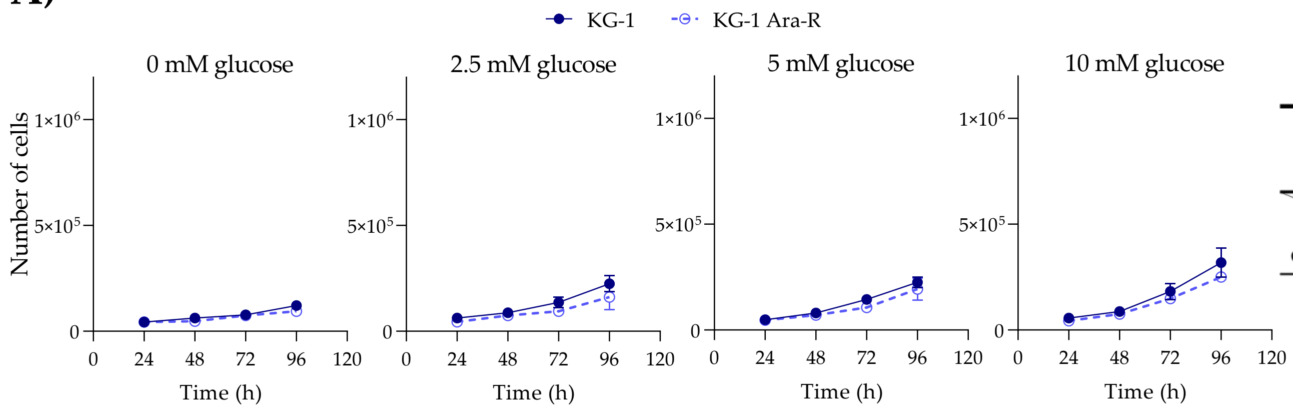

B)

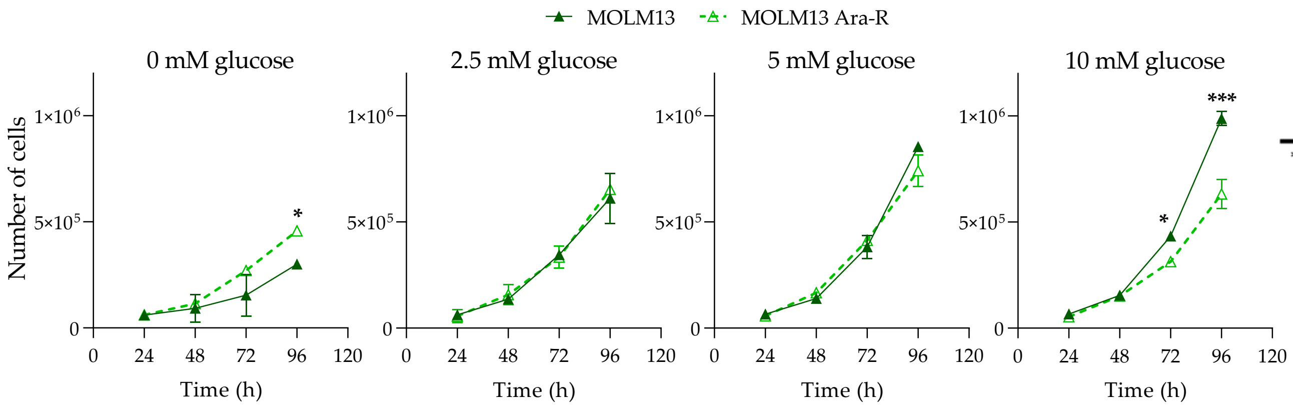

C)

Cell line \ Glucose (mM)	DOUBLING TIME (Td) ± SD (h)				GROWTH RATE (μ) ± SD (h^{-1})			
	0	2.5	5	10	0	2.5	5	10
**KG-1**	44.68 ± 3.05	32.61 ± 4.93*	30.44 ± 1.51*	26.67 ± 0.58*	0.014 ± 0.001	0.018 ± 0.006	0.021 ± 0.004	0.024 ± 0.002
**KG-1 Ara-R**	52.41 ± 5.57	38.40 ± 6.96	34.26 ± 1.51	28.40 ± 2.46*	0.011 ± 0.001	0.018 ± 0.002	0.020 ± 0.004	0.025 ± 0.007
**MOLM13**	25.15 ± 3.33	20.30 ± 1.63	18.54 ± 0.56	17.96 ± 0.53*	0.023 ± 0.005	0.032 ± 0.004	0.036 ± 0.003	0.038 ± 0.004*
**MOLM13 Ara-R**	26.34 ± 5.00	20.16 ± 0.58	19.33 ± 0.84	20.21 ± 0.42	0.028 ± 0.001	0.035 ± 0.008	0.036 ± 0.006	0.034 ± 0.001

* Significantly different comparing the different glucose concentrations with absence of glucose ($p \leq 0.05$).

Figure S3. Effect of different glucose concentrations on cell growth rates and doubling times of AML Ara-C resistant and parental cell lines. Cell growth curves of A) KG-1 and KG-1 Ara-R, and B) MOLM13 and MOLM13 Ara-R were analyzed over multiple population doublings. 2.5×10^4 cells were plated with 0, 2.5, 5 and 10mM glucose. Cells were counted every 24h using Trypan blue dye. C) Values of growth rates (μ) and doubling times (Td) were calculated from the respective line equations. Statistical significance was determined by two-way ANOVA followed by Sidak's Multiple Comparison Test. * $p \leq 0.05$; *** $p \leq 0.001$. At least three independent experiments with three replicates were performed.

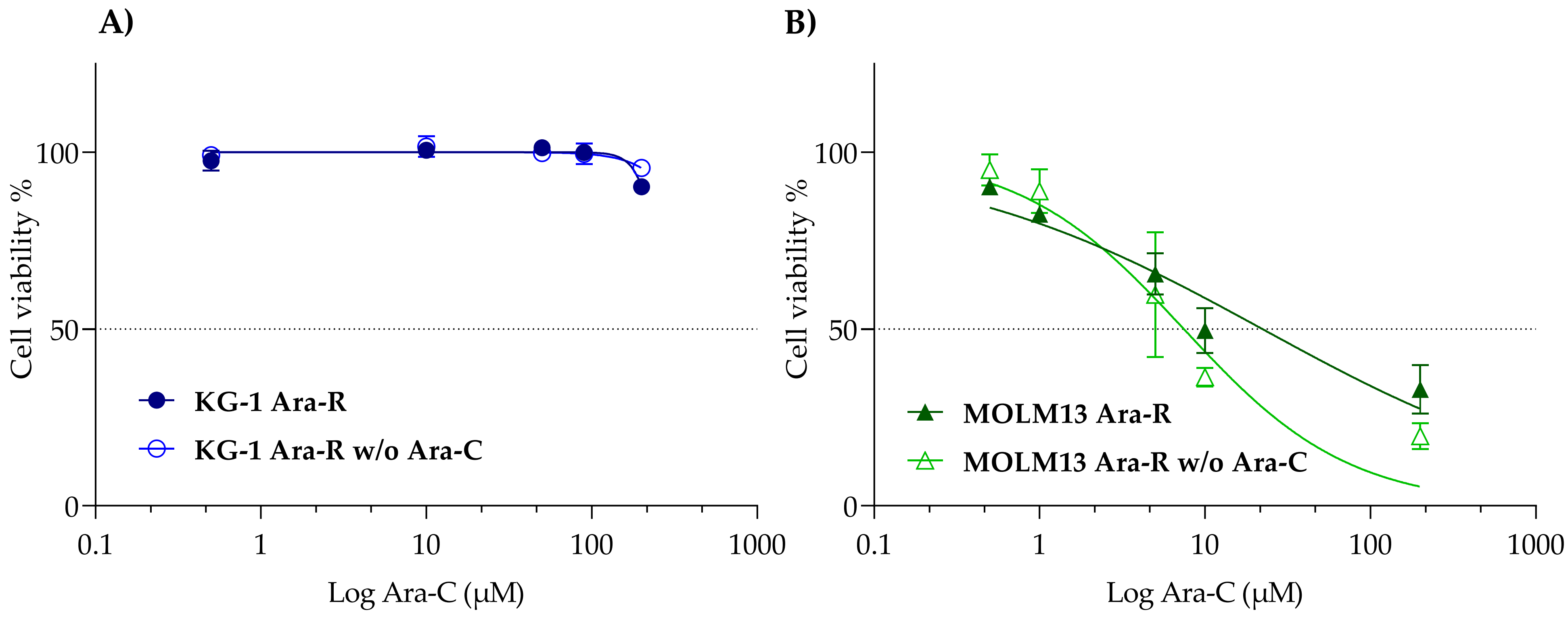

Figure S4. Sensitivity of cytarabine (Ara-C)-resistant cells in absence of Ara-C. Ara-C-resistant (A) KG-1 and (B) MOLM13 cells were cultured for three weeks with and without Ara-C. Cells were plated and tested with a range of Ara-C concentrations for 48h. Values are expressed as percentage of cell viability relative to vehicle-treated cells normalized to 100%. Values are given as mean ± SD. Two-way ANOVA followed by Sidak's Multiple Comparison Test. At least three independent experiments with two replicates were performed.

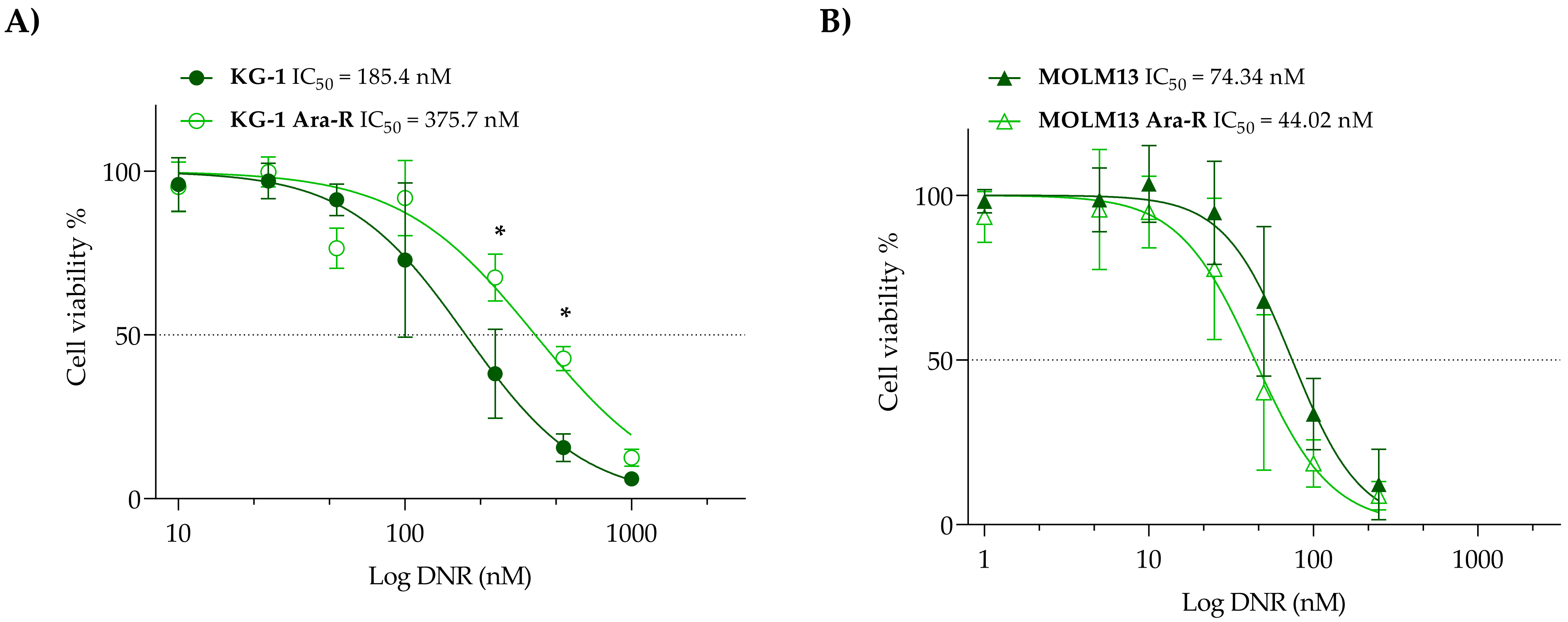

Figure S5. Sensitivity of cytarabine (Ara-C)-resistant cells to daunorubicin (DNR). Ara-C-resistant (A) KG-1 and (B) MOLM13 cells were plated and tested with a range of DNR concentrations. Values are expressed as percentage of cell viability relative to vehicle-treated cells normalized to 100%. Values are given as mean ± SD. Two-way ANOVA followed by Sidak's Multiple Comparison Test. At least three independent experiments with two replicates were performed. * $p \leq 0.05$.

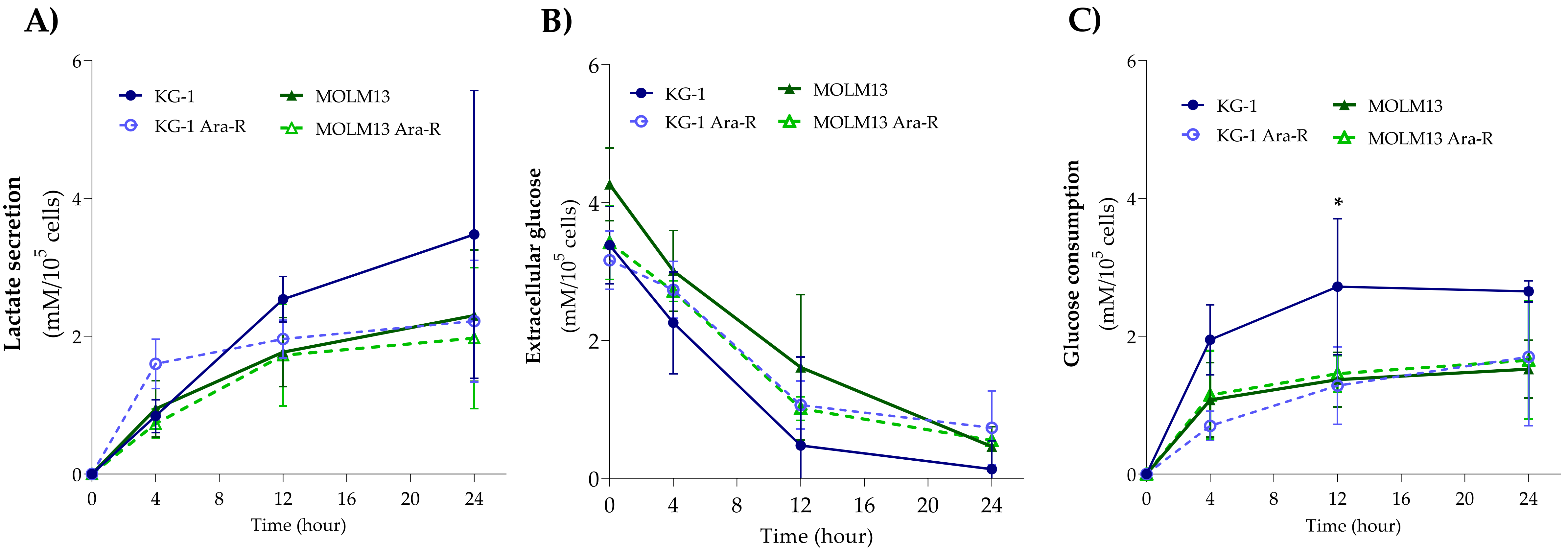

Figure S6. Extracellular lactate and glucose, glucose consumption in parental and Ara-C resistant cell lines. (A) Extracellular lactate levels and (B) extracellular glucose levels and (C) glucose consumption were evaluated at 0, 4, 12, and 24h for KG-1 and KG-1 Ara-R, and MOLM13, and MOLM Ara-R cells. Glucose consumption corresponds to the difference between 0 and 4, 12 or 24h of extracellular glucose levels. Results are presented as mean ± SD from three independent experiments. Statistical significance estimated by two-way ANOVA followed by Sidak's Multiple Comparison Test: *$p < 0.05$ (C).

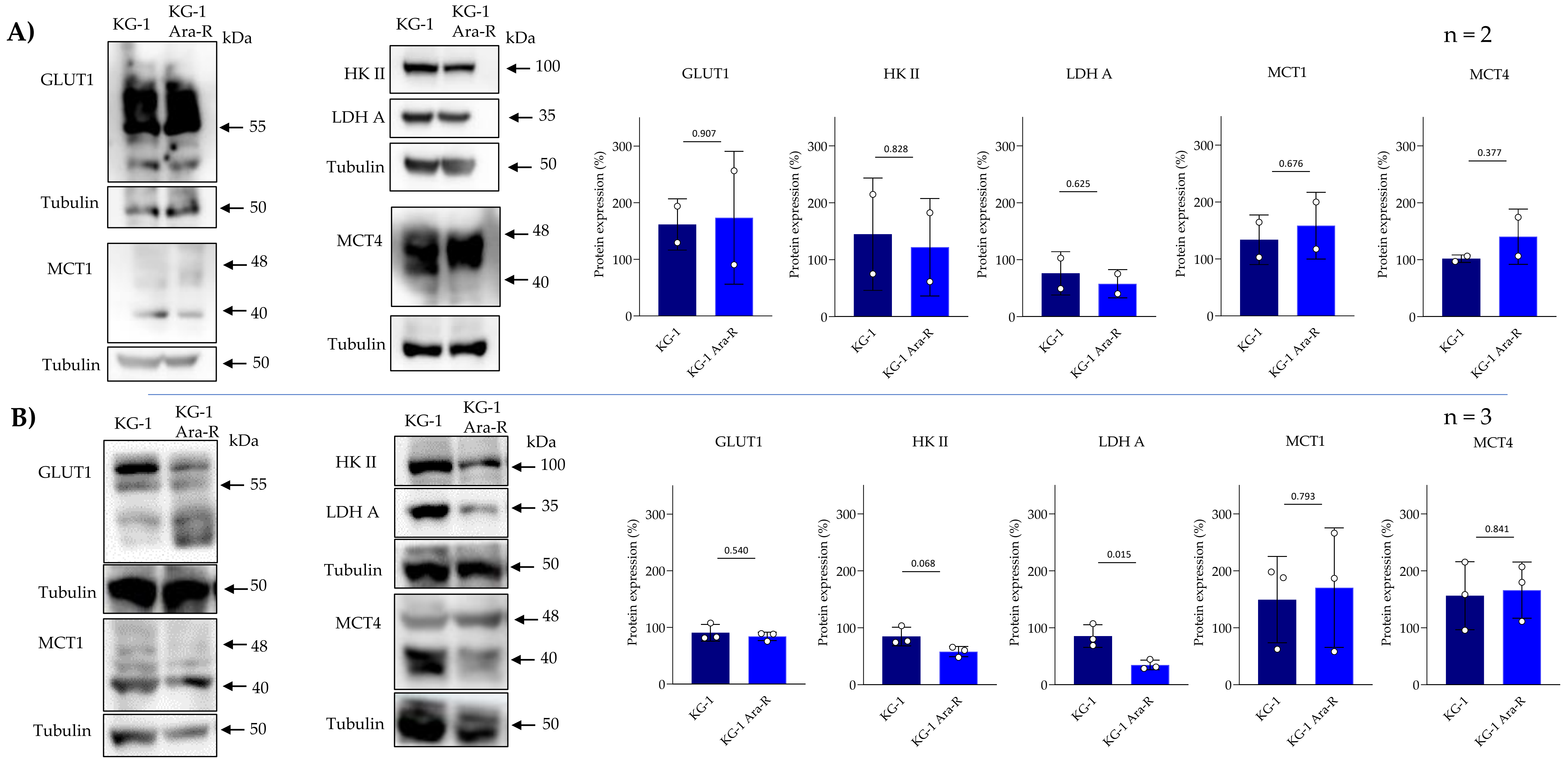

Figure S7. Characterization of glycolytic phenotype-associated protein expression in cytarabine (Ara-C)-resistant cell lines. Expression levels of GLUT1, HKII, LDHA, MCT1, and MCT4 proteins assessed by western blot in the cell pairs A,B) KG-1/KG-1 Ara-R and C,D) MOLM13/MOLM13 Ara-R cells at A,C) 6h and B,D) 24h. α-Tubulin was used as a loading control. Levels of protein expression were normalized for tubulin. Results were quantified using the ImageJ software and results are presented as mean ± SD of at least two independent experiments. Values were expressed as protein levels relative to parental cells normalized for 100%. Statistical significance was estimated by the unpaired two-tailed Student's t-test.

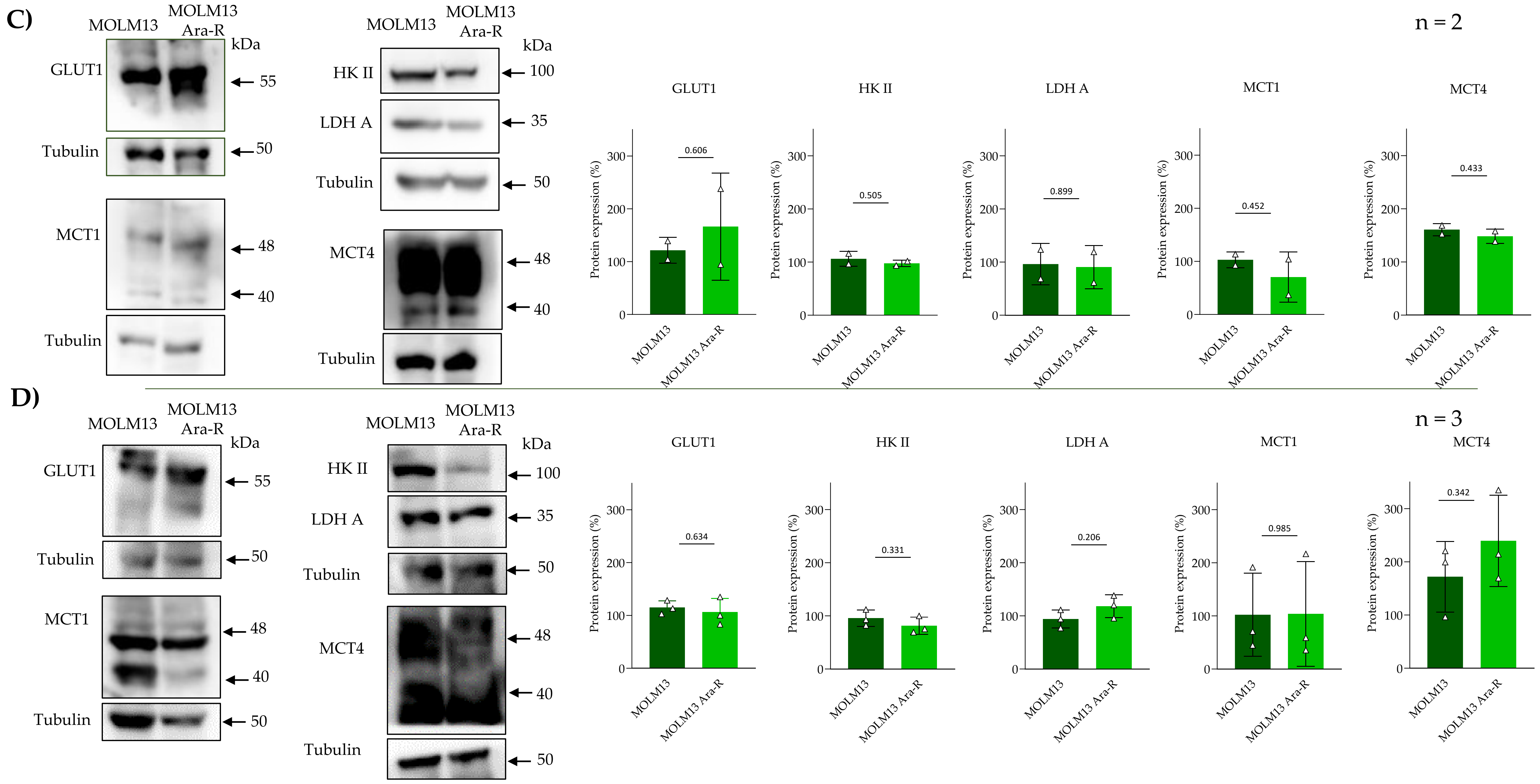

Figure S7. (continued).

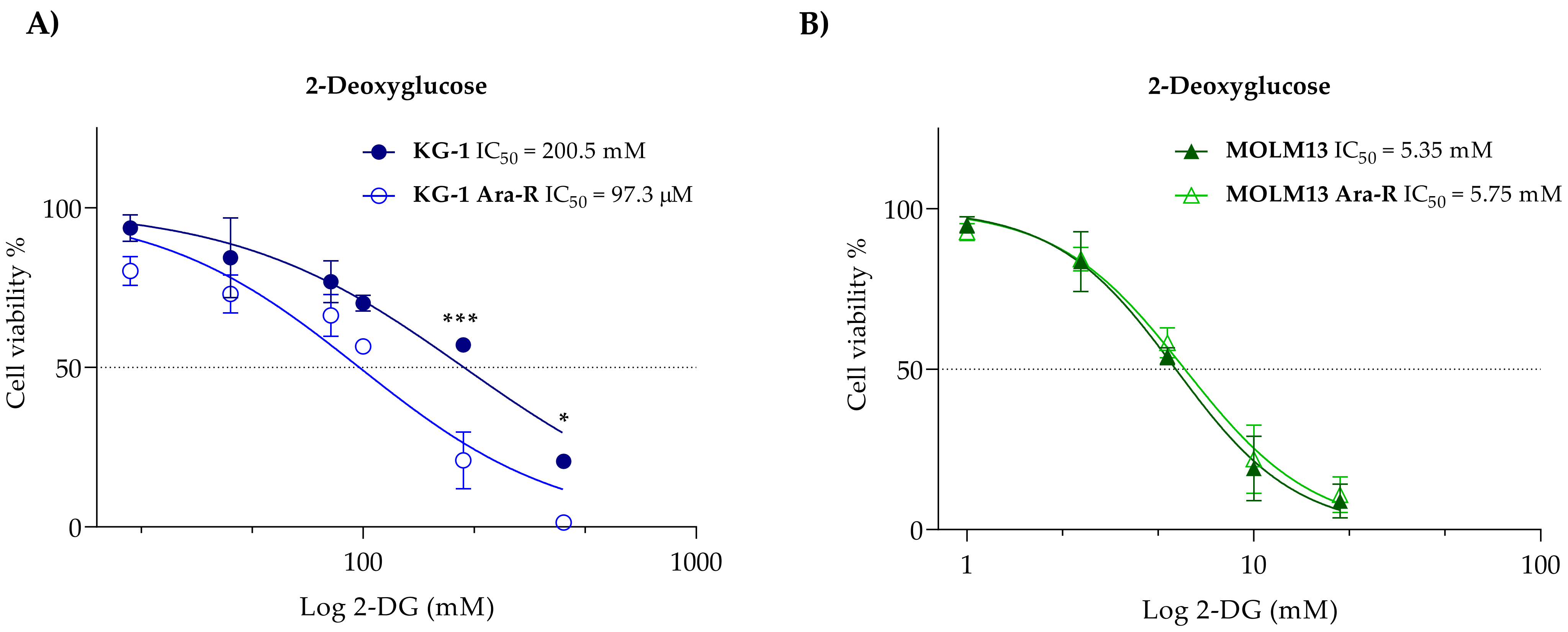

Figure S8. Effect of 2-deoxy-glucose (2-DG) on Ara-C-resistant and parental AML cell viability. Dose-response curve to generate IC_{50} values of (A) KG-1 and KG-1 Ara-R cell lines and (B) MOLM13 and MOLM Ara-R cell lines in response to 2-DG. Values are expressed as cell viability relative to vehicle-treated cells normalized to 100%. Values are given as mean ± SD. *$p < 0.05$; ***$p < 0.001$ (Two-way ANOVA followed by Sidak's Multiple Comparison Test). Results are from at least three independent experiments with two replicates.
